# Giant Hyalinizing Trabecular Tumor of the Thyroid: A Case Report and Literature Review

**DOI:** 10.2174/0115734056452029260623060307

**Published:** 2026-07-22

**Authors:** Caihong Wang, Fei Wang, Dan Qin, Yuyu Hua, Ying Yang, Yining Xiang, Jing Wen

**Affiliations:** 1 Academy of Medical Imaging, Guizhou Medical University, Guiyang, Guizhou 550004, China; 2 Department of Radiology, Luzhou People's Hospital, Luzhou 646000, China; 3 Department of Ultrasound Center, Affiliated Hospital of Guizhou Medical University, Guiyang, Guizhou 550004, China; 4 Department of Pathology, Affiliated Hospital of Guizhou Medical University, Guiyang, Guizhou 550004, China

**Keywords:** Hyalinizing Trabecular Tumor (HTT), Dual-Phase Contrast-Enhanced CT neck (DP-CECT neck), Ultrasound-Guided Fine-Needle Aspiration Cytology (US-FNAC), Arteriovenous Malformation (AVM), Severe exertional dyspnea, Inflammatory changes, Airway compression

## Abstract

**Introduction:**

Hyalinizing Trabecular Tumor (HTT) is an extremely rare thyroid neoplasm. Preoperative imaging diagnosis and Fine-Needle Aspiration Cytology (FNAC) are considerably challenging. We present a case of invasive HTT, describing its clinical course and imaging manifestations.

**Case Description:**

A 49-year-old woman presented with a 12cm left thyroid mass that had been incidentally detected as a 9×8mm nodule ten years previously. Following asymptomatic gradual enlargement, she developed severe dyspnea in March 2024, prompting admission. Physical examination confirmed a large, firm left thyroid mass. Ultrasound (US) was limited by the lesion's size. Dual-Phase Contrast-Enhanced CT (DP-CECT) revealed a 12cm oval mass replacing the left lobe. US-FNAC yielded suspicious for malignancy (TBSRTC Category III). She underwent left thyroid lobectomy and isthmusectomy. Pathology and immunohistochemistry confirmed HTT with focal capsular invasion. The patient recovered uneventfully during follow-up.

**Conclusion:**

Elucidating the characteristic features of HTT will facilitate early diagnosis and clinical intervention, providing valuable insights for future clinical research.

## 1 INTRODUCTION

HTT is a rare neoplasm originating from thyroid follicular epithelial cells, first described by Carney *et al*. in 1987 [1]. It typically exhibits non-invasive biological behavior, presents as a solitary lesion with a well-defined capsule, and is generally considered indolent. HTT shares morphological similarities with other thyroid pathologies, such as Papillary Thyroid Carcinoma (PTC), Medullary Thyroid Carcinoma (MTC), and Thyroid Paraganglioma (TPG), often leading to diagnostic confusion [2]. Intraoperative frozen section diagnosis is particularly challenging and prone to misinterpretation, underscoring the importance of accurate recognition.

The 4th edition of the World Health Organization Classification of Tumours of Endocrine Organs (2017) reclassified HTT as a borderline tumor, updating its ICD-O code from “0” (benign) in the 2004 edition to “1” (uncertain, indeterminate, or borderline malignancy) [3]. However, the 5th edition (2022) WHO Classification of Thyroid tumors replaced the “borderline” category with “low-risk tumor” [4]. Nevertheless, the malignant potential of HTT remains controversial, as it may still exhibit invasive behavior [2].

This article reports a case of a large HTT of the thyroid with a prolonged clinical course. The lesion was highly vascular, with an AVM identified within and around it. Histopathological examination revealed capsular invasion, suggesting aggressive biological behavior. The clinical progression and imaging features of this case are described in detail.

## 2 CASE PRESENTATION

### 2.1 Patient Information

A 49-year-old female farmer from rural southwestern China presented in March 2024 with severe exertional dyspnea. Ten years earlier, a 9×8 mm nodule had been incidentally detected in the left thyroid lobe during routine screening. As the lesion was asymptomatic, it remained untreated. The patient reported progressive enlargement of the mass over the preceding decade. Physical examination revealed a giant anterior neck mass obscuring the trachea. The overlying skin appeared normal without inflammatory changes. The mass was firm, non-tender, and extended retrosternally with an indistinct inferior border.

### 2.2 US Examination

The US revealed a large hypoechoic left thyroid mass exceeding the imaging field. The lesion demonstrated heterogeneous echotexture with multiple slit-like cystic spaces (Fig. **1A**, **B**). No calcifications were identified. Color Doppler Flow Imaging (CDFI) showed rich peripheral vascularity (Fig. **1C**). Per the Chinese Thyroid Imaging Reporting and Data System (C-TIRADS), the nodule was classified as category 4A.

### 2.3 Dual-Phase Neck CECT

The patient subsequently underwent DP-CECT of the neck. CT imaging revealed a giant oval mass in the left thyroid lobe, measuring approximately 12.0×7.7×5.9 cm. It extended from the lower border of the C3 vertebral body to the superior margin of the aortic arch, occupying most of the neck region. The margins were relatively well-defined, and no definite calcifications were identified within the lesion. In the arterial phase, the mass exhibited marked heterogeneous enhancement, with peripheral solid components and irregular patchy hypodense areas centrally. Notably, in the venous phase, the peripheral solid components showed significant centripetal filling, resulting in a marked reduction of the central hypodense area, which assumed a slit-like or stellate appearance. The mean CT attenuation values of the solid component were 149.74 HU in the arterial phase and 128.10 HU in the venous phase. (Fig. **2A**-**D**).

Using CT post-processing reconstruction techniques, Multiplanar Reformation (MPR), Maximum Intensity Projection (MIP), and Volume Rendering (VR) images were generated. The superior and inferior thyroid arteries bilaterally originated from the external carotid arteries and subclavian arteries, respectively. These vessels were significantly dilated and tortuous, coursing along the periphery of the mass to supply it, then penetrating into the lesion and anastomosing with central vessels. Drainage eventually occurred into the right external jugular vein and left common jugular vein, consistent with AVM formation. (Fig. **3A**-**F**).

### 2.4 Laboratory Examination

The patient's white blood cell count, neutrophil count, lymphocyte count, triiodothyronine (T3), thyroxine (T4), free triiodothyronine (FT3), free thyroxine (FT4), Thyroid-Stimulating Hormone (TSH), anti-thyroglobulin antibody (TGAb), anti-thyroid peroxidase antibody (TPOAb), thyroglobulin (Tg), and Carcinoembryonic Antigen (CEA) levels were all within normal ranges.

### 2.5 US-FNAC Examination

Ultrasound-guided FNAC of the left thyroid lesion revealed atypical follicular cells with nuclear enlargement, crowding, grooves, and microfollicular structures. Due to low cellularity and the absence of definitive features, a malignant diagnosis could not be established. Classified as TBSRTC III (Atypia of Undetermined Significance), further evaluation is required. (Fig. **4A**, **B**).

### 2.6 Postoperative Pathology

Due to significant airway compression by the large, hypervascular mass, the patient underwent surgery in late March 2024 following multidisciplinary consultation and exclusion of contraindications. After anesthesia induction and sterile preparation, intraoperative exploration revealed that the left thyroid was completely replaced by a solid, well-circumscribed 12×9 cm mass with markedly dilated and tortuous surface vessels. The inferior pole extended retrosternally, causing pronounced rightward tracheal deviation and deformation, though the tracheal rings remained intact without softening. No enlarged perithyroidal lymph nodes were identified. Following meticulous dissection and ligation of surrounding tissues and vessels, left thyroid lobectomy with isthmectomy was performed. Intraoperative frozen section examination, performed in conjunction with preoperative cytology, indicated a neoplasm of the left thyroid lobe and isthmus, with definitive classification pending permanent histopathology. The procedure was completed with cosmetic wound closure.

Immunohistochemical markers showed: CD56 (+), Ki-67 (approximately 1%+), CK19 (-), TPO (-), CgA (-), Syn (-), Galectin-3 (-), and calcitonin (-) . These findings supported the diagnosis of Hyalinizing Trabecular Tumor (HTT) in the left thyroid lobe with focal capsular invasion (Fig. **5A**-**D**).

### 2.7 Follow-up

The patient was prescribed levothyroxine sodium tablets postoperatively and recovered well overall. Follow-up evaluations were conducted in May 2024, September 2024, and February 2025 with the following results: FT3, FT4, and TSH levels remained within normal ranges. Thyroglobulin (Tg) levels were 4.09 ng/mL (normal range:3.50-77.0 ng/mL) in May 2024, decreased to 2.65 ng/mL in September 2024, and increased to 4.95 ng/mL (within normal range) in February 2025. Neck ultrasound examinations showed no significant abnormalities.

## 3 RESULTS OF THE LITERATURE REVIEW

Initially, a systematic search was conducted across the PubMed, Web of Science, and Embase databases, covering the period from January 2010 to January 2025. The search terms were as follows: (1) “Hyalinizing trabecular tumor of the thyroid” AND “ultrasonography”; (2) “Hyalinizing trabecular tumor of the thyroid ”AND(“computed tomography” OR“CT”); (3) “HTT” AND “ultrasonography”; (4) “HTT” AND (“computed tomography” OR “CT”). The flowchart of the literature screening process and the inclusion/exclusion criteria are presented in Fig. (**6**).

We focused on summarizing reports with imaging manifestations of HTT cases over the past 15 years (Table **1**) [5-18]: We ultimately included a total of 14 studies, summarizing the imaging features of 64 lesions. Most were case reports, with a few rare aggressive cases. HTT patients typically lack specific clinical symptoms and are predominantly identified incidentally. The age range was 22-81 years, with a higher prevalence in middle-aged and elderly females. Lesion sizes ranged from 0.2 to 5.5 cm and could occur in the left lobe, right lobe, and isthmus of the thyroid, primarily in the left and right lobes. All patients underwent preoperative conventional thyroid ultrasound, while a minority also underwent CT scans. Most patients were misdiagnosed with or suspected of having PTC based on FNAC findings. Surgical approaches mostly involved left or right lobectomy or total thyroidectomy. Several studies indicated that postoperative immunohistochemical detection of Ki-67 (MIB-1) is significant for diagnosing HTT. Patient postoperative follow-up ranged from 0.7 to 72 months, with no observed recurrence or metastasis.

## 4 DISCUSSION

### 4.1 Molecular Features and Immunohistochemistry of HTT

The PAX8-GLIS3 fusion represents the defining molecular event of HTT, detectable in 93~100% of cases [19, 20]. This fusion drives GLIS3 transcription factor overexpression, which upregulates collagen synthesis to form dense hyalinized stroma, physically restraining tumor growth, while concurrently activating Sonic Hedgehog (SHH) signaling to promote cell survival [19, 21]. Notably, PAX8-GLIS3 fusion is virtually absent in PTC, offering high specificity for differential diagnosis [19, 22].

Postoperative immunohistochemical staining for Ki-67 (MIB-1) in HTT frequently demonstrates a distinctive membranous/cytoplasmic positivity, in contrast to the nuclear staining observed in PTC. This aberrant localization appears highly characteristic of HTT and aids in its differentiation from PTC [22]. In differential diagnosis, HTT typically shows negative or only focal weak expression of CK19 and Galectin-3, whereas these markers are usually diffusely and strongly positive in PTC. The utility of HBME-1 is less consistent, with some studies reporting negative expression in HTT while others have detected positivity in a subset of cases [23, 24].

### 4.2 Clinical and Imaging Manifestations of HTT

This retrospective study revealed the following findings [5-18]: HTT predominantly occurs in middle-aged and elderly women, often incidentally detected as thyroid nodules during physical examinations, with some patients presenting as cervical masses and disease duration extending up to 15 years. Tumor diameters range from 0.2-5.5cm; smaller lesions may be misinterpreted as vascular structures on ultrasonography, while larger ones occasionally exhibit microcalcifications. Owing to substantial cytomorphological overlap with PTC, the diagnostic accuracy of cytology is approximately 8%. Surgical management primarily comprises lobectomy or total thyroidectomy, with no recurrence or metastasis observed during postoperative follow-up ranging from 0.7-72 months. Although capsular invasion may occur in HTT, no reliable reports of lymph node metastasis currently exist, a stark contrast to PTC, which frequently demonstrates irregular margins, microcalcifications, capsular disruption, and regional lymph node metastasis. Ultrasonographically, HTT typically presents as solitary, solid, hypoechoic, well-circumscribed ovoid nodules with an anteroposterior-to-transverse ratio <1; cystic clefts are occasionally observed, whereas calcifications are rare. Color Doppler imaging frequently demonstrates rich peripheral and internal vascularity. Nevertheless, definitive diagnosis by conventional ultrasound remains challenging. Jang and Lee [8, 11] noted that HTT lacks malignant features on ultrasound evaluation, whereas Choi *et al*. [6] reported that approximately 29% of HTT cases may exhibit malignant sonographic characteristics. Currently, CT studies on HTT remain scarce, with prior descriptions characterizing these lesions as well-defined, regular hypodense masses. The present study further observed that on contrast-enhanced CT, HTT may exhibit centripetal filling of peripheral solid components during the venous phase, with central cystic regions compressed into slit-like configurations. Although solid components maintain relatively high CT values in the venous phase, their enhancement remains inferior to normal thyroid parenchyma, with increased, tortuous vessels forming arteriovenous malformations within and around the lesion. These imaging features likely result from the trabecular architecture of HTT tumor cells and the abundant hyaline material and mucoid matrix within the stroma, which retard contrast agent diffusion through the extravascular interstitium, yielding delayed enhancement. Owing to slow growth and firm consistency secondary to hyalinization, cystic areas in HTT do not develop the large, rounded cavities characteristic of PTC, but are instead compressed by surrounding dense neoplastic tissue into irregular slit-like or stellate configurations. Conversely, PTC typically demonstrates irregular morphology, ill-defined margins, marked heterogeneous enhancement with rapid washout on contrast-enhanced CT, clearly distinguishing it from HTT. However, the absence of enhancement curves or time-density analysis in this study represents a limitation that will guide the design of more comprehensive protocols in future investigations.

### 4.3 Surgical Decision-Making and Perioperative Risks

Intraoperative examination in this case revealed severe tracheal compression with rightward deviation and deformation, though the tracheal rings remained intact without softening, accounting for the patient's severe dyspnea. For giant HTT, awareness of potential tracheal compression or softening is paramount, as anesthesia induction carries a risk of airway collapse, necessitating thorough preoperative assessment and contingency planning. Given the hypervascular nature and potential vascular malformations, blood products should be on standby, and controlled hypotension should be considered intraoperatively. Additionally, intraoperative neuromonitoring is essential to preserve the recurrent laryngeal nerve and parathyroid function, highlighting the critical importance of multidisciplinary collaboration in developing individualized therapeutic strategies.

### 4.4 Future of AI and Radiomics in HTT

Given the high susceptibility of HTT to misdiagnosis on conventional imaging and cytological examinations, artificial intelligence-assisted diagnostic techniques demonstrate substantial potential. Recent studies indicate that AI classifiers integrating hand-crafted radiomic features with high-dimensional features extracted through deep learning have achieved exceptional performance, even surpassing experienced radiologists in diagnostic efficacy [25]. Similar radiomics workflows facilitate the extraction of subtle textural and vascular patterns imperceptible to less experienced clinicians. Such AI-assisted tools serve not to replace pathological diagnosis but to provide a critical decision support system, particularly valuable in settings with limited subspecialty expertise in thyroid imaging.

## CONCLUSION

Although HTT is a low-risk neoplasm, large tumors may cause airway compression and bleeding, necessitating multidisciplinary collaboration for individualized perioperative management to balance precise treatment against overtreatment. Radiomics and artificial intelligence play crucial roles in differential diagnosis, representing important future research directions.

## Figures and Tables

**Fig. (1):**
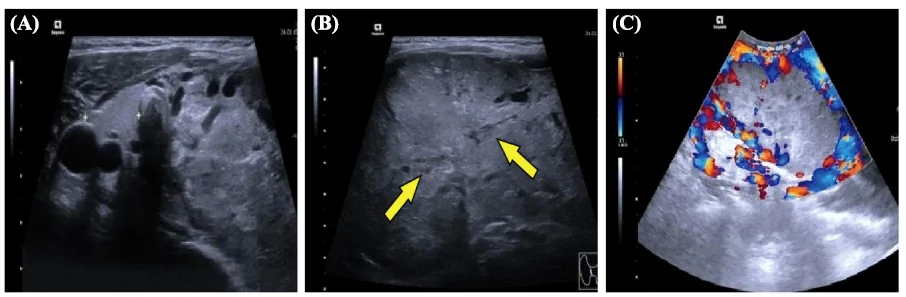
Ultrasonography: A massive lesion was observed in the left lobe of the thyroid gland, the extent of which was difficult to accurately delineate (**A**). Multiple scattered slit-like cystic hypoechoic areas were noted within the lesion (yellow arrows, **B**). Color Doppler flow imaging demonstrated prominent peripheral vascularity surrounding the lesion (**C**).

**Fig. (2):**
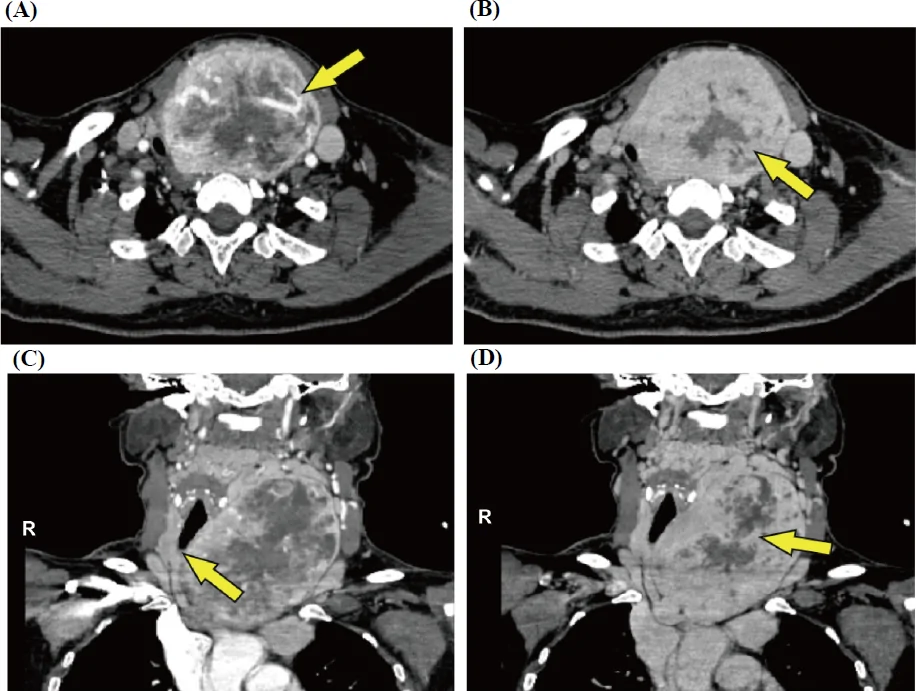
Contrast-enhanced CT images. (**A**, C: arterial phase; **B**, D: venous phase). During the arterial phase, the lesion demonstrates heterogeneous enhancement with the presence of enlarged, tortuous intralesional vessels (yellow arrows, A). Significant compression and flattening of the main bronchus is observed (yellow arrow, **C**). In the venous phase, the solid components show progressive fill-in enhancement compared to the arterial phase, with the central low-attenuation areas appearing slit-like and stellate (yellow arrows, B, **D**).

**Fig. (3):**
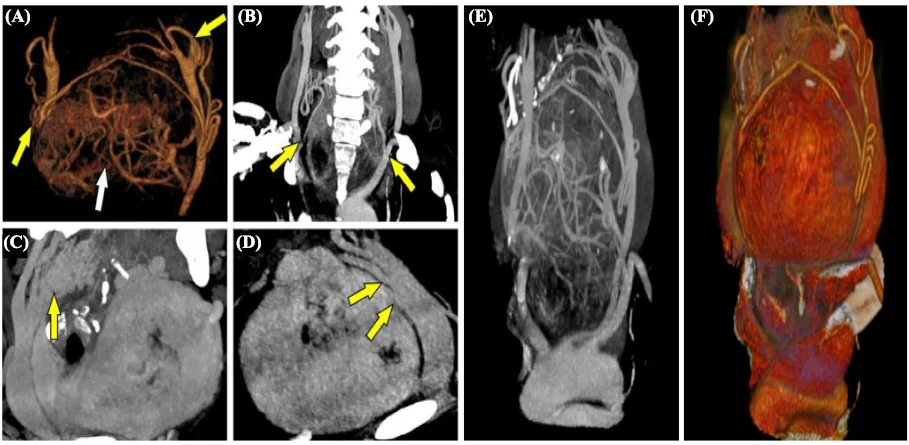
Postprocessed CT images (VR, MPR, MIP): (**A**, **B**) Feeding arteries: bilateral superior and inferior thyroid arteries (yellow arrows); (**A**) intratumoral central vessels (white arrow); (**C**, **D**) Drainage veins: a single confluence into the right external jugular vein (**C**) and two confluences into the left common jugular vein (**D**) (yellow arrows); (**E**) Overall MIP image demonstrates the relationship between the lesion and the surrounding vasculature. (**F**) Overall VR image displays the relationship between the lesion and the adjacent vessels.

**Fig. (4A, B):**
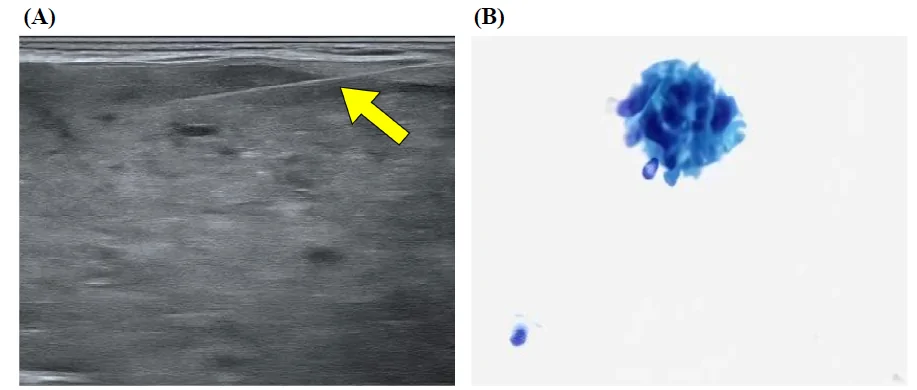
Ultrasound-guided Fine-Needle Aspiration Cytology (FNAC): Cells suspicious for malignancy were observed (TBSRTC: The Bethesda System for Reporting Thyroid Cytopathology, Category III).

**Fig. (5):**
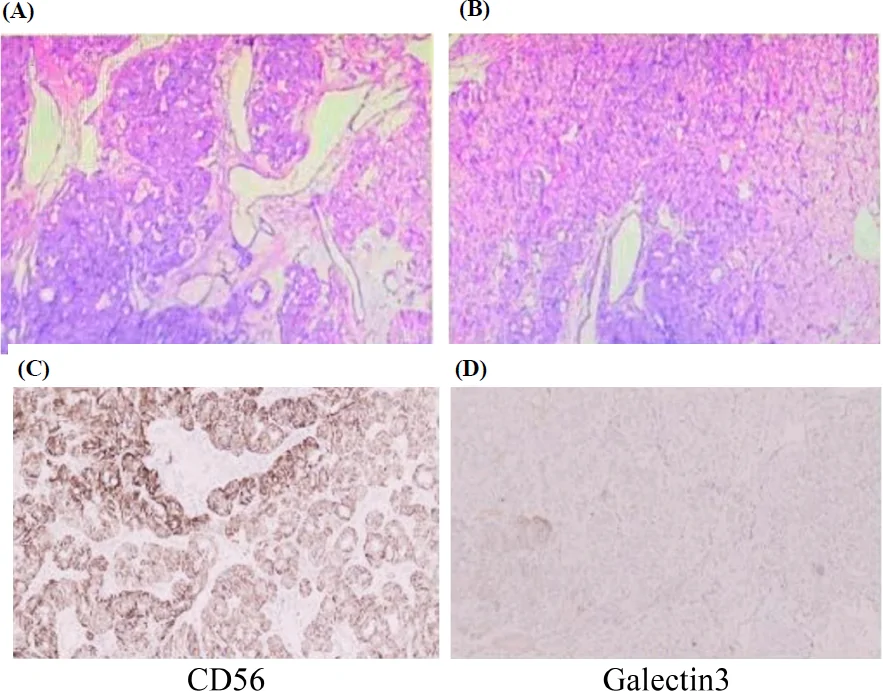
(**A**, **B**) Histopathological examination with HE staining: A tumor in the left thyroid lobe and isthmus is observed, requiring immunohistochemistry for further diagnosis; (**C**) CD56 shows positive expression; (**D**) Galectin3 shows negative expression. HTTImmunohistochemical profile: CD56(+), Ki-67(approximately 1%+), CK19(–), TPO(–), CgA(–), Syn(–), Galectin3(–), Calcitonin(–).

**Fig. (6):**
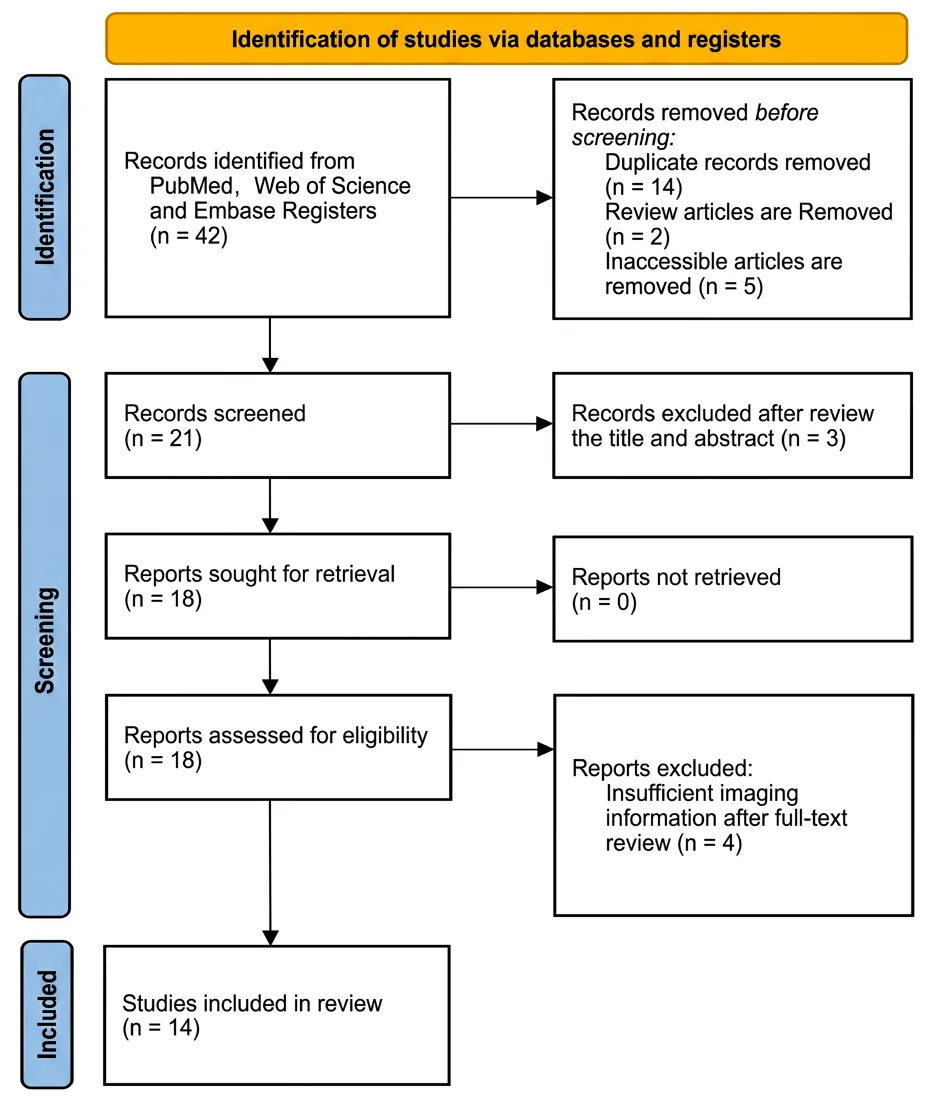
Literature screening flowchart.

**Table 1:** Clinical and imaging data of 15 HTT cases reported from 2010 to 2025.

**Case No.**	**Reference**	**Nationality**	**Symptom**	**Age** **(years)**	**Sex**	**Location**	**Number (cases)**	**Size** **(cm)**	**Imaging**		**Type of resection**	**Follow-up** **(months)**	**FNAC**	**Pathology**	**Immunohistochemistry**
									**Ultrasonograph**	**Computed tomography/CT**					**ki-67**
1	Lee S [11] (2011)	Korea	Screening(5/10);Grave’s disease(2/10);Hoarseness(1/10); Neck swelling(1/10);Palpable nodule(1/10)	26~81	9F/1M	6L/4R	10	0.6~4.2	Solid texture (10/10), oval to round shape (10/10), a well-defined aspect (10/10), hypoechoic character (8/10), heterogeneous character (7/10), the presence of a hypoechoic halo (8/10), and no microcalcifications (10/10)	NA	TT,CND(7/10);LL, isthmusectomy(1/10); RL,isthmusectomy(1/10); Hemithyroidectomy(1/10)	28;30;8;11;7;NA(5/10)	PTC(6/10);Suspicious for PTC(2/10);PTC *vs*. HTT(2/10)	HTT	NA
2	Choi WJ [6] (2015)	Korea	NA	23~58	21F/3M	NA	24	NA	All nodules were oval-to-round;87.5% (21/24)had a smooth margin, 75% (18/24) were hypoechoic or markedly hypoechoic, and 91.7% (22/24) were solid. CDUS results were available for 17 patients, and all showed peri- and/or intranodular vascularity. Seven patients had malignant US tumor features, such as marked hypoechogenicity or a spiculated margin. None of the patients had a tumor, with a taller-than-wide shape or calcification	NA	NA	NA	AUS(5/24);FLUS(1/24);Suspicious for PTC(9/24);PTC(2/24);MTC(1/24);CNB results HTT(4/24);Incidental OP finding HTT(4/24);ND(1/24)	HTT	The membranes of tumor cells were immunoreactive for MIB-1
3	Jang H [8] (2016)	Korea	Palpable-mass(1/12);Hypothyroidism(1/12); Negative(10/12)	44~70	11F/1M	NA	12	0.3~4.1	Hypoechogenicity or marked hypoechogenicity (83.4%); Absence of calcifications (91.7%); Parallel shape (100.0%), presence of vascularity (75.0%), and probable benignity (58.3%)	NA	Subtotal(2/12); Hemitotal(6/12);Total(4/12)	72;10;22;13;18;21;31;17;55.9;NA(3/12)	Suspicious for PTC(3/12);PTC(3/12);PTC *vs*. HTT(2/12);Follicular neoplasm(1/12);AUS/FLUS(1/12); NA(1/12)	HTT	ki67(+)(7/12);Ki67(-)(2/12);NA(3/12)
4	Liu Y [13] (2017)	China	Pharynx discomfort(1/4);A palpable mass for ten years(1/4);Incidentally detected(2/4)	45~51	3F/1M	3L/1R	4	0.4~5.0	All of the patients showed solitary hypoechoic nodules with abundant blood flow signals	There was a clear low-density shadow in the affected thyroid lobe(3/4). One case showed multiple plaques of a large mass and displayed displacement of the trachea due to compression of a large mass; NA(1/4)	Hemitotal(3/4); Subtotal(1/4)	30;36;6;10	1NA;1AUS/FLUS Suspicious for PTC;2Suspicious for PTC	HTT	Ki-67(Clone:MIB-1):showed membranous/cytoplasmic immunoreactivity; Ki-67(Clone:SP6)(<1%)
5	Tajiri K [16] (2017)	Japan	Incidentally discovered	44	F	R	1	0.2	A hypoechoic nodule located in the right lobe, it was oval in shape and isolated. The margin was well-defined but slightly rough. It was not associated with a hypoechoic halo. The echotexture was homogeneous. There was no calcification. The Power Doppler image showed intense intratumoral blood flow	NA	Total thyroidectomy	NA	NA	HTT	The cell membrane was positive for Ki-67(MIB1, 1:200 dilution, DAKO)
6	Jones DJ [9] (2017)	USA	Incidentally discovered	70	F	R	1	1.35×1.94×1.32	Mildly heterogeneous and hyperechoic nodule	NA	Total thyroidectomy	1	AUS	HTT	Ki-67 (MIB-1) stains were negative
7	Rhee YY [15] (2018)	Korea	Medical checkup discovered	63	F	L	1	0.6×0.4	The lesion was well circumscribed, a benign feature, although it did show hypoechoic features with internal microcalcification	Revealed a low-density nodule with no evidence of extracapsular invasion and no significant lymph node enlargement	Left lobectomy	NA	Showed features of PTC	HTT	Diffuse membrane positive reactivity for Ki-67 (MIB1 clone) at room temperature
8	Katano H [10](2021)	Japan	Painless, elastic, left cervical mass	54	F	L	1	4.1×4.5×2.4	A heterogeneous mass showing a well-defined border, with a cystic internal lesion, was identified in the left lobe of the thyroid gland	The mass showed a low density with enhancement similar to that of the thyroid parenchyma, except in the cystic portion. In addition, the trachea was displaced to the right by the tumor	NA	18	Chronic thyroiditis on two occasions, and the possibility of malignancy could not be ruled out out	HTT	Ki-67 was positive for cytoplasm, and negative for the cell membrane, and the nucleus was nearly negative
9	Xing YJ [18] (2022)	China	Mobile with swallowing was found	47	F	R	1	3.0×2.0×4.5	Hypoechoic mass with clear boundary, aspect ratio <1, uneven internal echo, abundant blood flow signal, resistance index of 0.64, and TI-RADS class of 4a	NA	Right thyroidectomy	NA	NA	HTT	NA
10	Alsogair O [5](2023)	SAU	Presented with anterior neck swelling	60	M	R	1	0.7×0.8	A small, rounded, calcified nodule near the isthmus	NA	Total thyroidectomy	0.7	Suspected to be malignant	HTT	Thyroid membrane and peripheral cytoplasm reactivity(brown color) of Ki-67 immunohistochemistry staining
11	Lukas J [14] (2023)	Czech Republic	NA	48	M	I and R	1	2.7×2.5×3.0	A voluminous hypoechogenic nodule with numerous minor calcifications and increased vascularization was found in the isthmic part and right lower lobe, reaching the upper edge of the sternum. According to ATA, high node suspicion, classification TI-RADS 4c	Enlarged isthmus of the thyroid gland 21–26 mm, which reaches the edge of the sternum with an inhomogeneous nodule and calcifications	Total thyroidectomy	NA	Bethesda III-AUS	HTT	Ki-67(MIB1)-membrane positivity
12	Hayashi S [7] (2024)	Japan	Incidentally discovered	43	F	L	1	0.8×0.7×0.7	A well-defined, hypoechoic mass with microcalcifcation in the left lobe of the thyroid gland	Solid mass exhibiting slight homogeneous enhancement in the lower pole of the thyroid gland	Left hemithyroidectomy	10	AUS	HTT	Weak membranous and cytoplasmic staining was found by MIB1 (Ki-67) immunostaining
13	Liu C [12] (2025)	China	Ultrasound examination discovered	60	F	L	1	5.5×4.1×3.3	A well-defined, hypoechoic mass with microcalcification in the left lobe of the thyroid gland	Enlargement of the left lobe of the thyroid with a mass-like low-density shadow inside. In addition, the left lobe exhibited poorly defined borders and an uneven density with visible calcifications inside the mass	Total thyroidectomy	6	NA	HTT	Positivity for Ki-67 was <5% of tumor cells
14	Wang F [17] (2024)	China	A routine physical examination(5/5), while one patient had a neck mass for 15 years, and this mass was enlarged within 1 year before hospital admission	22~51	4F/1M	3L/2R	5	0.8~5.0	Hypoechoic nodule with clear boundary and high blood flow signals(5/5)	NA	NA	60;42;36;24;10	HTT(2/5);suspicious PTC(3/5)	HTT	Ki-67(MIB-1)(+)
15	Our case	China	Dyspnea	49	F	L	1	12.0×7.7×5.9	A slightly hypoechoic mass, which was large and unmeasurable, with multiple areas of fine fissure-like cystic degeneration, surrounded by abundant blood flow signals	Uneven density, occupying a large part of the left side of the neck, with clear borders, and with no calcification seen within it. In the arterial phase, the lesion shows obvious uneven enhancement, with a solid periphery and an irregular, flaky hypointense area in the center. In the venous phase, the solid part is obviously filled inward, and the central hypointense shadow is obviously narrowed down to show fissure-like and stellate changes, and the enhancement of the lesion seems to be eosinophilic tumor changes	Left hemithyroidectomy	6	AUS	HTT (invasive)	Ki-67 (about 1%+)

**Abbreviations:** F, female; M, male; L, Left lobe of the thyroid gland; R, Right lobe of the thyroid gland; I, Isthmus; NA, Not available; FNAC, fine needle aspiration cytology; AUS, atypia of undetermined significance; FLUS, Follicular Lesion of Undetermined Significance; ND, Non-Diagnostic.

## Data Availability

The data and supportive information are available within the article.

## LIST OF ABBREVIATIONS

HTT: Hyalinizing trabecular tumor
PTC: Papillary thyroid carcinoma
FNAC: Fine-needle aspiration cytology
US: Ultrasound
DP-CECT: Dual-phase contrast-enhanced computed tomography
MPR: Multiplanar reformation
MIP: Maximum intensity projection
VR: Volume rendering

## STANDARDS OF REPORTING

CARE guidelines were followed.
